# A novel P300 BCI speller based on the Triple RSVP paradigm

**DOI:** 10.1038/s41598-018-21717-y

**Published:** 2018-02-20

**Authors:** Zhimin Lin, Chi Zhang, Ying Zeng, Li Tong, Bin Yan

**Affiliations:** 1 China National Digital Switching System Engineering and Technological Research Center, Zhengzhou, China; 2 Key Laboratory for NeuroInformation of Ministry of Education, School of Life Science and Technology, University of Electronic Science and Technology of China, Chengdu, China

## Abstract

A brain–computer interface (BCI) is an advanced human–machine interaction technology. The BCI speller is a typical application that detects the stimulated source-induced EEG signal to identify the expected characters of the subjects. The current mainstream matrix-based BCI speller involves two problems that remain unsolved, namely, gaze-dependent and space-dependent problems. Some scholars have designed gaze-independent and space-independent spelling systems. However, this system still cannot achieve a satisfactory information transfer rate (ITR). In this paper, we propose a novel triple RSVP speller with gaze-independent and space-independent characteristics and higher ITR. The triple RSVP speller uses rapid serial visual presentation (RSVP) paradigm, each time presents three different characters, and each character is presented three times to increase the ITR. The results of the experiments show the triple RSVP speller online average accuracy of 0.790 and average online ITR of 20.259 bit/min, where the system spelled at a speed of 10 s per character, and the stimulus presentation interface is a 90 × 195 pixel rectangle. Thus, the triple RSVP speller can be integrated into mobile smart devices (such as smartphones, smart watches, and others).

## Introduction

A brain-computer interface (BCI) system based on electroencephalography (EEG) is a popular research direction in the field of human-computer interaction. The system can provide a direct communication channel to connect the human brain and computer. An EEG speller system is a typical brain-computer interface system. The speller system uses a clever paradigm to induce specific event-related potential (ERP) components (for example, P300 component). Then, according to the ERP components, a symbol of the expected subjects can be determined. The EEG speller system can re-establish the disabled communication, and normal people can use the technology to obtain convenient interactive methods^1–6^. At present, most EEG speller systems are based on modified P300 speller. The P300 component is a common ERP component, which shows a peak when small probability events are observed after approximately 300–500 ms^7–10^. The P300 component also exhibits significant waveform characteristics in the time domain^11^. A P300 detection algorithm is essential because it determines the accuracy and reliability of BCI systems. Thus, some scholars have attempted and proposed many P300 detection algorithms such as independent component analysis (ICA)^12^, common spatial pattern (CSP)^13^, xDawn^14,15^, hierarchical discriminant component analysis (HDCA)^16–19^, sliding HDCA (sHDCA)^20,21^, and convolutional neural network (CNN)^22^. On this basis, many BCI system performances were improved significantly.

Farwell and Donchin first proposed P300 alphabet speller system (FD speller)^23^. The system is a 6 × 6 matrix, and each element is a specified character. The system has a total of 36 characters (26 letters and other control characters) and uses a stepwise linear discriminant analysis (SWLDA) algorithm to detect P300 components. Krusienski *et al*.^24^ compared the performances of various P300 detection algorithms and concluded that SWLDA and Fisher’s linear discriminant (FDA) are suitable for the P300 Speller system. In the FD speller, the matrix rows or columns blink randomly, and when the locked symbol is hit by rows or columns (probability is 1/6), it induces a P300 component. The FD speller determines the expected symbol position using the detected P300 component. Cuntai Guan *et al*.^25^ believed that if the probability of a character being hit is small, then the P300 component induced is evident and detection is easier. Thus, Guan *et al*. proposed a single-character display random flashing speller system (SC speller) based on the FD speller (the probability that the character was hit was 1/36). Furthermore, Townsend *et al*.^26^ proposed a checkerboard paradigm speller system, that is, a random flash hits multiple characters, and then a special coding is employed to confirm the anticipant character of the subject. This method can achieve higher accuracy and mean bit rate. Brian Roark *et al*.^27,28^ proposed Huffman scanning, which uses Huffman coding to select the symbols to highlight, based on the FD speller. Qi LI *et al*.^29^ proposed a familiar face (FF) spelling paradigm, which introduces a familiar face to improve recognition accuracy based on the FD speller. They further proposed a green familiar face (GFF) paradigm^30^. Dewen Hu *et al*.^31^ proposed a hybrid BCI speller that fuses P300 with steady-state visual evoked potential (SSVEP) and introduced an SSVEP feature on P300-row and P300-column feature. Chen *et al*.^32,33^ used the SSVEP feature and introduced the phase feature to build a high-speed speller to achieve the highest information transfer rate (approximately one character per second). These speller systems are matrix-based spellers.

In the last two decades, research on the EEG speller has shown exciting achievements, but many problems remain unsolved. Brunner *et al*.^34^ showed that FD speller-based recognition accuracy is more dependent on the gaze of the subject; it requires the user to not move his eyes, and then often cannot achieve the desired accuracy. Therefore, the matrix-based speller has a gaze-dependent feature. This condition limits the use of the matrix-based speller in some patients with severe neuromuscular disability. Brendan *et al*.^35^ showed that the larger the matrix, the better the performance. Salvaris *et al*.^36^ demonstrated that when the character is small, the performance is worse. Therefore, the matrix-based speller has a space-dependent feature. The matrix-based speller cannot easily produce sufficiently small results that people between the mild disabled and normal cannot easily spread.

To solve these problems, many scholars have proposed non-matrix structure speller paradigm. Gabriel *et al*.^37^ proposed a GIBS block speller that effectively overcomes the problem of the matrix-based speller. Treder *et al*.^38^ proposed an ERP Hex-o-Spell paradigm, a two-level speller consisting of six discs arranged on an invisible hexagon. The method can achieve higher accuracy than the FD speller when eye movements are not permitted. Fabio Aloise *et al*.^39^ proposed a Geometric Speller to optimize the design of Hex-o-Spell. Treder *et al*.^40^ compared the Hex-o-Spell, Cake Speller, and Center Speller. The three different variants of a two-stage visual speller are based on covert spatial attention and non-spatial feature attention. The results show that they can achieve higher accuracy and independent of eye gaze. However, this method is still not sufficiently convenient. Orhan *et al*.^41–43^ proposed RSVP keyboard, using rapid serial visual presentation (RSVP) paradigm for spelling. In the RSVP paradigm, each candidate letter is shown at the same place on the screen in a temporally ordered sequence at a comfortably high presentation rate. Chennu *et al*.^44^, Moghadamfalahi *et al*.^45^, and Acqualagna *et al*.^46,47^ showed that the EEG classifiability with the RSVP speller was as good as that with the FD speller, and the RSVP speller has gaze-independent and space-independent characteristics. Therefore, the RSVP speller can be used by persons with sight disabilities, and the design of the interface can be extremely small. The RSVP speller has a tempting potential to be integrated into small intelligent devices (such as smartphones, augmented reality devices, and virtual reality devices). However, relative to most matrix-based spellers, the RSVP Speller information transfer rate (ITR) is not satisfactory. Literature^44,45^ indicates that the RSVP speller (which contains 28 characters) has only one-third of the ITR of the Matrix speller (which contains 36 characters). Therefore, improving the ITR of the RSVP speller system under the condition of ensuring RSVP speller recognition accuracy is an extremely meaningful subject of study.

The typical RSVP speller presents a scene (a character) at a time. If we present multiple characters at a time, then we can effectively shorten the time to display all characters, and then enhance the ITR. We propose a triple-RSVP speller. The system uses the RSVP paradigm and shows three different characters, with each character appearing three times. We asked the subjects to observe the three characters at the same time. Finally, we group average the corresponding EEG signal for each character, and determine the P300 component and expected characters of the subject.

## Results

### Visual stimuli and procedure

**Figure 1:**
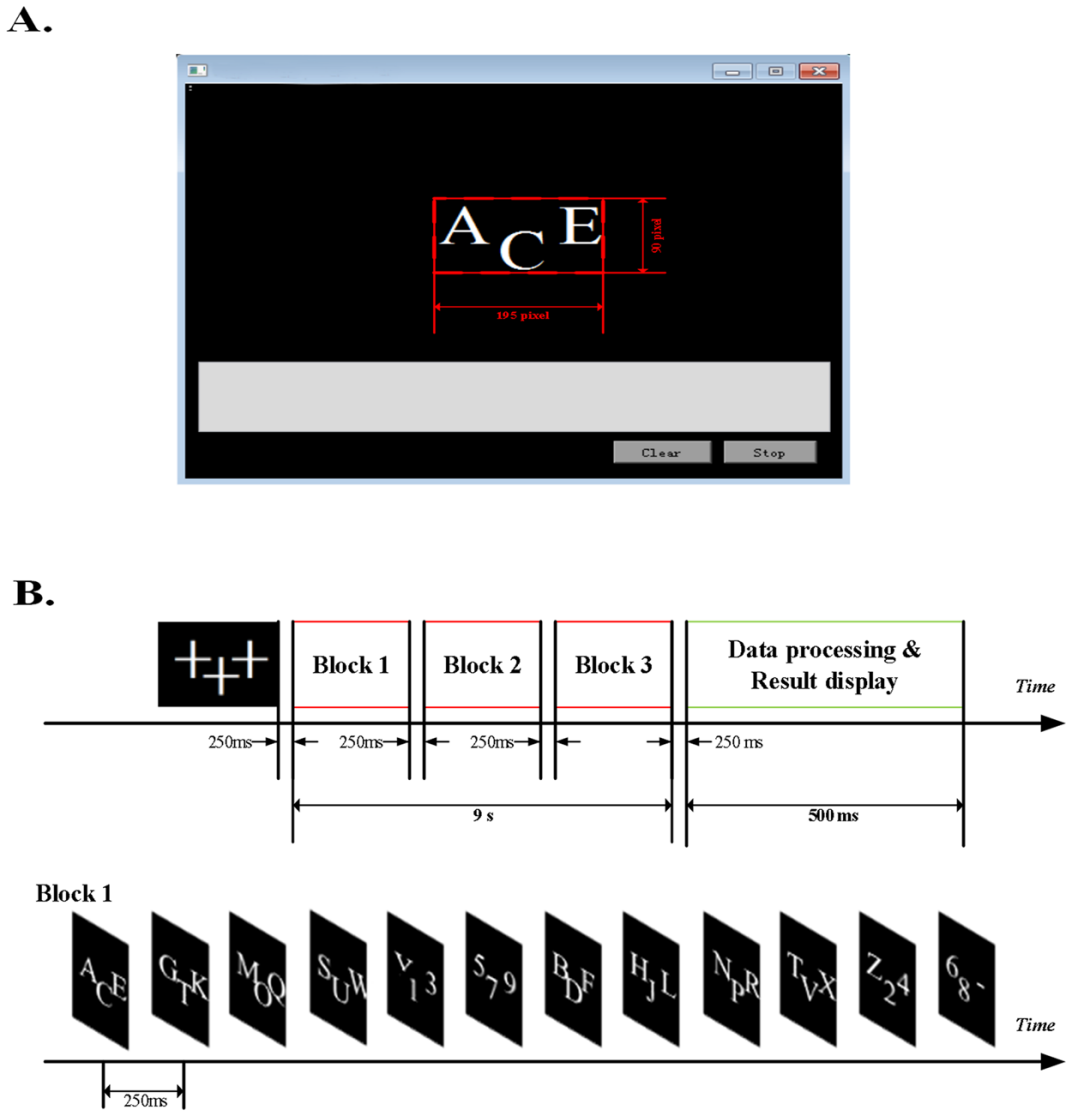
Stimulus presentation interface of triple RSVP speller.

**Figure 2:**
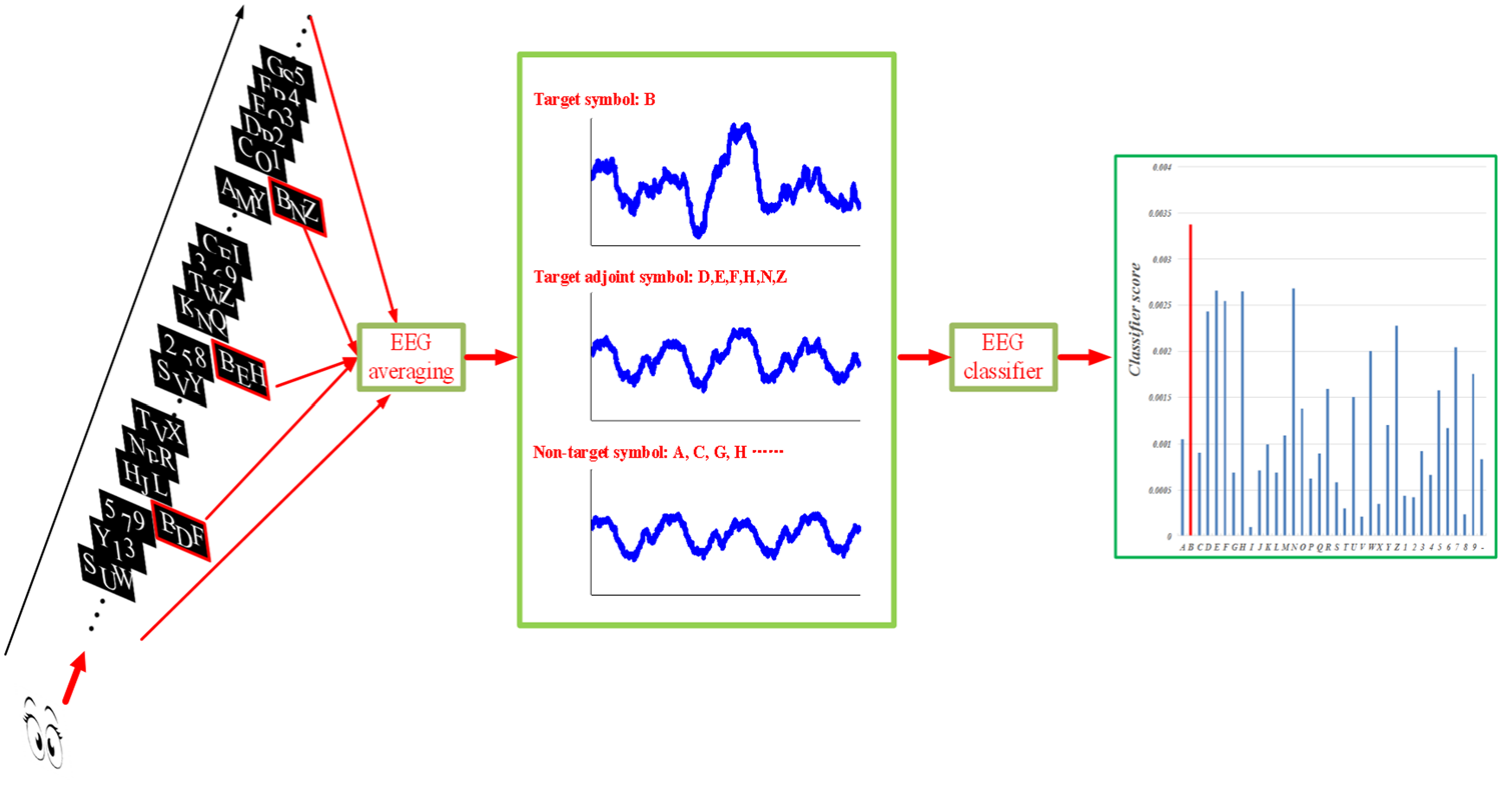
Triple RSVP speller system overview. The subject looks at the symbol group sequence consisting of three blocks, and focus on the specific character, for example “B”. In the triple RSVP speller, each trial shows three characters, and each character appears three times. The EEG signal that corresponds to each character is group averaged, and the score is calculated by a trained P300 classifier. Finally, the highest score of all symbols is identified as the target character for the subjects.

The triple-RSVP speller presentation interface is shown in Fig. 1(A). The three symbol presentation areas are rectangles with size of 60.00 × 65.00 pixels, parallel arranged, and the middle symbol presentation area is offset down by 30 pixels to ensure that subjects can view three characters at the same time, in a short time. We use a 17.3-inch mobile workstation screen with a screen resolution of 1920 × 1080 pixels. Every 250 ms (4Hz), the three symbols in presentation areas refresh once at the same time. The new symbol is presented in three presentation areas. The symbol group presentation order is determined by the symbol group sequence, such as block 1, block 2, and block 3. Each block contains 36 characters, each three characters make up a symbol group, and each symbol group appears in three symbol presentation areas. Thus, each block contains 12 symbol groups (36 characters make up 12 symbol groups and each symbol group contains 3 characters). The three blocks are different, and the symbol group and positions of the different blocks are specially designed to ensure that the position of each character appears unique in different blocks. The subjects must watch three blocks to identify an expected character. During this period, the expected character appears three times in different symbol groups of different blocks. We average the EEG to correspond to the each character, determine the P300 component, and identify the expected symbol, as shown in Fig. 2.

As shown in Fig. 2, the subject looks at the symbol group sequence that consists of three blocks, each block consists of 12 symbol groups (12 trials), each trial shows three characters, and each character appears three times. For example, when the target character is “B,” the subjects notice three symbol groups: “BDF” in block 1, “BEH” in block 2, and “BNZ” in block 3. Each symbol group has a corresponding EEG signal; thus, the character of the same symbol group corresponds to the same EEG signal. Therefore, we designed unique symbol groups in three blocks. When we group average the EEG signals of a character, the P300 component of the target character can be easily detected. For example, in Fig. 2, we group average the EEG signal of symbol group ”BDF”, ”BEH”, and ”BNZ” as the group average signal of character “B”. Similarly, the group average signal of non-target character is composed of the EEG of all symbol groups that contain the non-target character, and the target adjoint character is also true. The symbol groups of the target adjoint character contain the target character; thus, the group average EEG of the target adjoint character contains some of the P300 component. For example, the group average EEG of character “D” consists of “BDF,” “ADG”, and “DP2”, where “BDF” contains the target character B. Thus, a trial P300 component is introduced during the mean processing of the letter “D” to allow the classifier score of the target adjoint character to be closer to the target character score. The design details of the symbol group sequence are in the Methods section.

### Event-related responses

**Figure 3:**
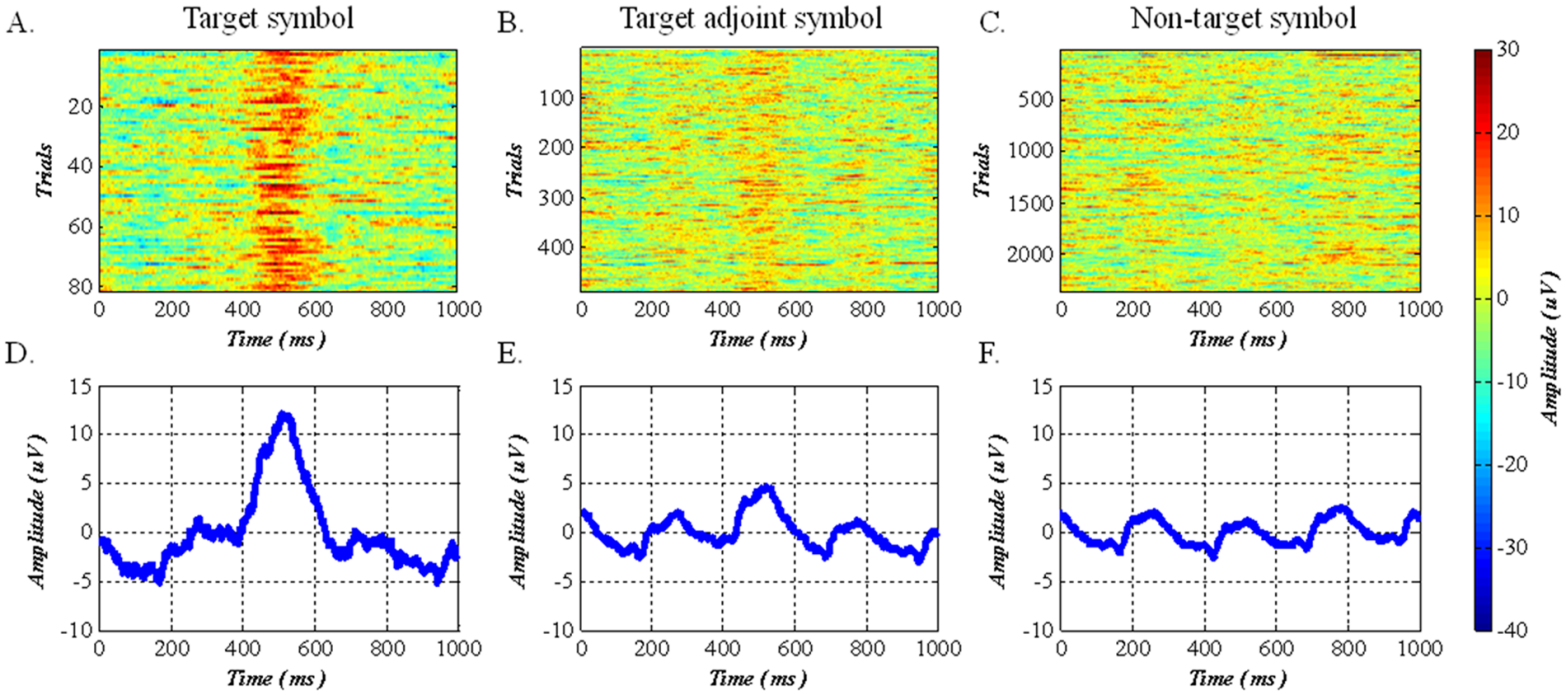
EEG signals of different categories (target, target adjoint, and non-target symbol), measured by electrode Pz. (**A**) The target character corresponds to the mean of the EEG signal. Each line represents the group average of the EEG signals of the target character (three symbol groups, each symbol group has a trial EEG, and the signal of the target character is the mean of the three symbol group), and the color indicates the signal amplitude value. Similarly, (**B)** and (**C**) indicate the cases of target adjoint symbol and non-target symbol, respectively. (**D**) indicates the averages across all signals of (**A**). (**E**) indicates the averages across all signals of (**B**). (**F**) indicates the averages across all signals of (**C**).

We calculate the mean of the signal corresponding to the target character, target adjoint character, and non-target character, as shown in Fig. 3(A,B and C), respectively. An evident peak is observed in the mean signal corresponding to the target character in Fig. 3(A), between 400 and 600 ms. The target adjoint character has an undetectable peak between 400 and 600 ms because the three symbol groups that correspond to the target adjoint character contain a target character. Therefore, the P300 component is included in the corresponding EEG signal of a symbol group, resulting in the target adjoint character mean EEG signal containing some of the P300 components. In addition, the P300 component is not included in the signal of a non-target character. All categories of the signals contain 4 Hz harmonic components because of the symbol group presentation speed of 4 per second.

### Triple-RSVP speller performance

This study tested the triple RSVP speller using a copy spelling task. Table 1 lists the selected accuracy and ITR for all subjects. The triple RSVP speller offline average accuracy is 0.780, and the average ITR is 19.876 bit/min. The online average accuracy is 0.790, and the average ITR is 20.259 bit/min. The system spelled at a speed of 10 s per character, and the stimulus presentation interface is a 90 × 195 pixel rectangle.

**Table 1:** Classification accuracy (%) and ITR (bit/min) in triple RSVP speller.

Subject	Accuracy, %		ITR, bit/min	
	Training (offline)	Testing (online)	Training (offline)	Testing (online)
sub1	0.742	0.732	18.137	17.740
sub2	0.812	0.831	21.050	21.886
sub3	0.663	0.702	15.117	16.575
sub4	0.663	0.732	15.117	17.740
sub5	0.756	0.783	18.701	19.813
sub6	0.951	0.922	27.819	26.248
sub7	0.733	0.716	17.780	17.114
sub8	0.821	0.883	21.443	24.295
sub9	0.779	0.785	19.646	19.897
sub10	0.828	0.814	21.753	21.137
sub11	0.776	0.753	19.521	18.579
sub12	0.715	0.741	17.075	18.097
sub13	0.902	0.882	25.228	24.247
Mean	0.780	0.790	19.876	20.259
SD	0.084	0.071	3.665	3.101

## Discussion

We propose a novel triple RSVP speller with two notable features as follows: gaze-independent and space-independent features. The current mainstream BCI speller system is often based on the matrix structure. This structure of the speller system can achieve a higher information transfer rate. However, two major problems are not resolved, namely, gaze-dependent and space-dependent problems. The gaze-dependent problem refers to the subjects that must look at specific clues when spelling a character. Thus, when spelling a character in a covert attention approach, satisfactory select accuracy and information transfer rate are often not achieved. The space-dependent problem refers to the stimulation presentation interface that requires a large screen. Some studies have shown that in the matrix-based speller, when the matrix achieved is large, the performance is better; when the character is small, the performance is worse. Thus, the matrix-based speller cannot use a smaller screen (for example, a mobile phone screen) to ensure a certain accuracy and information transfer rate conditions; it is detrimental to BCI speller spread among normal people.

In the face of gaze dependent issue, many scholars have proposed covert attention speller systems such as the GIBS block speller^37^, Hex-o-spell^38^, Geometric Speller^39^, and RSVP speller^42,43,48^. However, these methods cannot achieve a satisfactory information transfer rate. The GIBS block speller, Hex-o-spell, and Geometric Speller in the design cannot solve the problem of space dependency. Compared with these speller systems, we designed the triple RSVP speller system that can achieve a higher information transmission rate.

**Figure 4:**
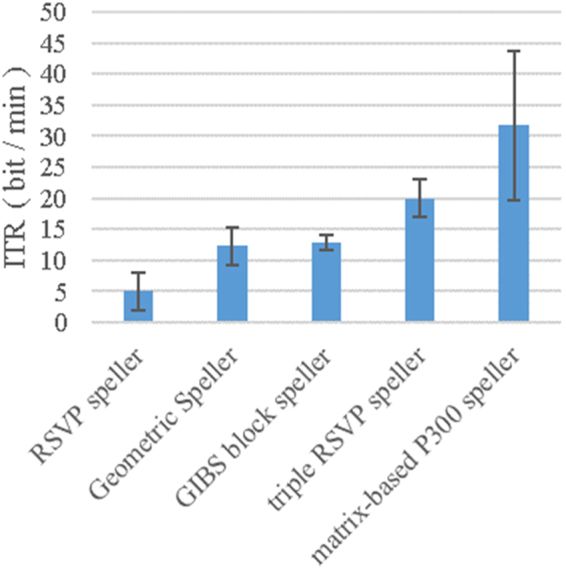
Information transfer rates for different speller systems. The RSVP speller, Geometric speller, GIBS block speller, and triple RSVP speller are gaze-independent spelling system. The matrix-based P300 speller refers to the current typical P300 spelling system to achieve the ITR level.

As shown in Fig. 4, we compared the triple RSVP speller with the mainstream gaze-independent spell system (the RSVP speller, Geometric speller, and GIBS block speller, and the data are derived from references^44,39^, and^37^, respectively) and the matrix-based P300 speller (data are derived from literature^31^) information transfer rate.

The RSVP keyboard was proposed by Orhan *et al*.^41–43^, using rapid serial visual presentation (RSVP) paradigm for spelling. In the RSVP paradigm, each candidate letter is shown at the same place on the screen in a temporally ordered sequence at a high presentation rate, however, the ITR of this method is not satisfactory. The Geometric Speller was proposed by Aloise *et al*.^39^, a total of *N*^2^ characters are grouped into 2N sets of N characters (analogous to rows and columns of the FD speller). In this arrangement, each character belongs to exactly two sets. In the visual layout of each set, characters are displayed at the vertices of a regular geometric figure. During the presentation, each set of characters is displayed transiently on the screen. The GIBS block speller was proposed by Gabriel *et al*.^37^, the symbols are grouped into four blocks following an alphabetical order. This layout is composed by a group of 9 symbols in the center and 3 lateral small blocks with the remaining symbols. To select a symbol, the user has first to select the small block where it belongs. When the respective block is selected, the symbols of that block move to the center and the respective small block disappears. The Geometric speller and GIBS block speller have higher ITRs, but require larger screen space. The matrix-based P300 speller is a classic BCI system, but the gaze-dependent and space-dependent problems cause it cannot be widely used in severe neuromuscular disability and normal people.

In this paper, the proposed triple RSVP speller proposed has space-independent features. Compared to the matrix-based BCI speller, the triple RSVP speller can be integrated in a small screen. In this experiment, we reduced the stimulus presentation interface to a size of 195 * 90 pixels. Compared to that of the matrix-based spelling system, the RSVP sequence is less demanding to stimulate size and spatial location requirements. Thus, the proposed triple RSVP speller can be embedded in the vast majority of portable smart devices (such as smartphones, smart watches, and others). The speller is useful for the BCI technology spread in daily life, and disabled persons can conveniently use it.

We propose the triple RSVP speller with gaze-independent and space-independent characteristics. Compared to the traditional gaze-independent spelling system, the triple RSVP speller can achieve higher letter spelling speed on a smaller screen. However, compared to the matrix-based spelling system, the triple RSVP speller ITR is still relatively low. Our goal is to improve the triple RSVP speller ITR.

## Methods

### Symbol group sequence

**Figure 5:**
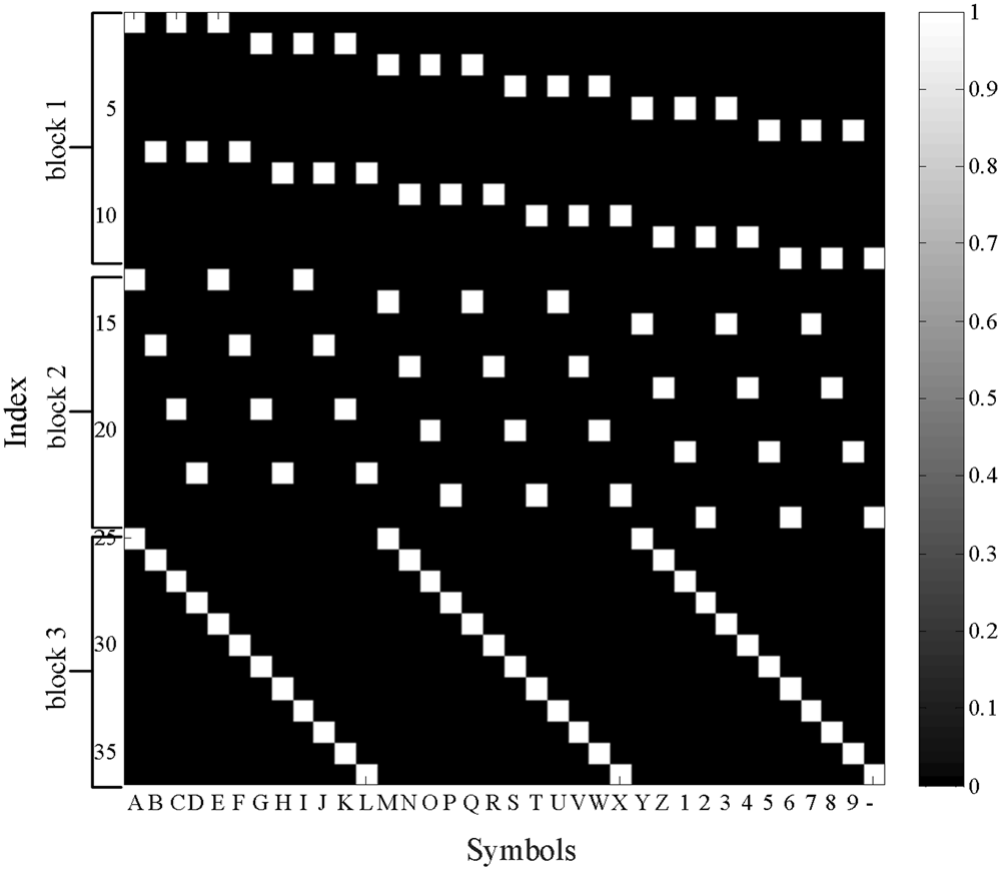
Rule of symbol group sequence. The abscissa represents 36 characters, and the ordinate represents the order in which the characters are presented, where 1 to 12 are block 1, 13 to 24 are block 2, and 25 to 36 are block 3. The symbol group presented in different trials is composed of white-marked characters.

The design of the symbol group sequence in the proposed triple RSVP speller is crucial. Our design criteria are as follows. First, each symbol must be presented again at a longer time interval. Thus, we designed three blocks, and each block contains the same 36 characters. Second, the symbol group of each character is different in different blocks to avoid confusion with the target character and the target adjoint character when distinguishing the classifier score. We designed the symbol group sequence as shown in Table 2 and Fig. 5.

**Table 2:** Symbol group sequence.

Index	1	2	3	4	5	6	7	8	9	10	11	12
block1	ACE	GIK	MOQ	SUW	Y13	579	BDF	HJL	NPR	TVX	Z24	68-
Index	13	14	15	16	17	18	19	20	21	22	23	24
block2	ADG	JMP	SVY	258	BEH	KNQ	TWZ	369	CFI	LOR	UX1	47-
Index	25	26	27	28	29	30	31	32	33	34	35	36
block3	AMY	BNZ	CO1	DP2	EQ3	FR4	GS5	HT6	IU7	JV8	KW9	LX-

### Participants

A total of 13 subjects (11 males and 2 females, average age of 22.1 ± 1.5 years, and right-handed) participated in the experiment. All subjects were students of Zhengzhou University and did not have any previous training in the task. The subjects exhibited normal or corrected-to-normal vision with no neurological problems and were financially compensated for their participation. This study was conducted after we obtained informed consent and Ethics Committee approval of China National Digital Switching System Engineering and Technological Research Center, and was carried out in accordance with the approved guidelines. All of the participants provided their written informed consent to participate in this study.

### EEG acquisition

EEG data were acquired by a g.USBamp system (G.Tec), using 16 electrodes distributed in accordance with the international 10–20 system. The electrooculographic (EOG) activity were recorded by two electrodes positioned above and below the left eye. We collected a group of EOC samples before the experiment and implemented the EOC artifact removal by using the method proposed by Chi Zhang^49,50^.

The EEG data were sampled at 2400 Hz using 200 Hz low-pass and 50 Hz notch filters. Prior to scoring the images, we pre-processed the EEG data through the following steps: downsampling to 600 Hz, band-pass filtering (0.1–60 Hz), and baseline correction. Zero- delay filtering was implemented using the filtfilt() function in MATLAB. Then, the EEG data were divided into epochs. Each epoch consisted of 1000 ms of EEG data after the stimulus onset.

### EEG data analysis

Analysis of the ERP using HDCA algorithm was performed as described by Parra *et al*.^16,17,51^. The HDCA algorithm can be divided into two layers. First, the HDCA algorithm was employed to obtain the average data and divide the original EEG data by time window size. The weight of each channel was then calculated in each time window to maximize the differences between the target and non-target classes. The time window size cannot be determined in advance. Thus, we selected 25 ms as the time window size after numerous experimental repetitions. The weight of each channel in each time window was calculated by Fisher linear discriminant (FLD). In each time window, the EEG signal was reduced to one dimension, such as in Equation (1), as follows:

1 $${y}_{k}=(\frac{1}{N})\sum _{n}\sum _{i}{w}_{ki}{x}_{i[(k-1)N+n]},$$

where ${x}_{i[(k-1)N+n]}$ represents the *k*th separate time-window value from the single-trial data. The variable corresponds to the EEG activity at the data sample point *n* measured by electrode *i*. *w* is a set of spatial weights. Weight vector *w*_*ki*_ is identified for the *k*th window and *i* electrode following each image presentation (*T* is the temporal resolution of the time window, which is 0.025 in this paper, *N* is the sampling time point of the time window, *F*_*S*_ is the sampling rate, *K* is the number of time windows, and $n=1,2,\cdots ,N,\quad N=T\times {F}_{S}$, $0\le k\le K$). *y*_*k*_ is the signal after reduced dimension in *k*th separate time-window. The time window size cannot be determined in advance. Thus, we selected 25 ms as the time window size after numerous experimental repetitions. The weight of each channel in each time window was calculated by Fisher linear discriminant (FLD).

2 $${y}_{IS}=\sum _{k}{v}_{k}{y}_{k},$$

The results for the separate time windows (*y*_*k*_) were then combined in a weighted *y*_*k*_ average to provide a final interest score (*y*_*IS*_) for each image. FLD analysis was used to calculate the spatial coefficient *w*_*ki*_, and logistic regression was adopted to calculate the temporal coefficient *v*_*k*_, such as in (2).

The time window size is 25 ms, *k* = 40.

### Evaluation of triple RSVP speller performance

To evaluate the performance of our triple RSVP speller, we computed the classification accuracy and ITR, which is widely used in the BCI speller community^52^. The ITR is given by

3 $$ITR=\frac{({\mathrm{log}}_{2}N+P{\mathrm{log}}_{2}P+(1-P){\mathrm{log}}_{2}\frac{1-P}{N-1})}{Time},$$

where *Time* is the time interval per selection. In this paper, we designed the triple RSVP speller to select a character that takes 10 seconds. *N* is the number of possible choices, and *P* is the probability that the desired choice will be selected in a process called target identification accuracy or classifier accuracy.

In the performance evaluation stage, we calculated the precision accuracy and information transfer rate of all subjects in the offline and online environments, respectively. In the training phase, we asked the subjects to focus on specific characters and collected EEG data for offline analysis and P300 classifier training. Then, we asked the subjects to spell a series of specific characters in the copy mode to calculate the online accuracy and ITR.
